# Choice of Moisturiser for Eczema Treatment (COMET): feasibility study of a randomised controlled parallel group trial in children recruited from primary care

**DOI:** 10.1136/bmjopen-2016-012021

**Published:** 2016-11-16

**Authors:** Matthew J Ridd, Kirsty Garfield, Daisy M Gaunt, Sandra Hollinghurst, Niamh M Redmond, Kingsley Powell, Victoria Wilson, Richard H Guy, Nicola Ball, Lindsay Shaw, Sarah Purdy, Chris Metcalfe

**Affiliations:** 1 Centre for Academic Primary Care, School of Social and Community Medicine, University of Bristol, Bristol, UK; 2 Bristol Randomised Trials Collaboration, School of Social and Community Medicine, University of Bristol, Bristol, UK; 3 Department of Pharmacy and Pharmacology, University of Bath, Bath, UK; 4 Department of Dermatology, University Hospitals Bristol NHS Foundation Trust, Bristol, UK

**Keywords:** PRIMARY CARE

## Abstract

**Objectives:**

To determine the feasibility of a randomised controlled trial of ‘leave on’ emollients for children with eczema.

**Design:**

Single-centre, pragmatic, 4-arm, observer-blinded, parallel, randomised feasibility trial.

**Setting:**

General practices in the UK.

**Participants:**

Children with eczema aged 1 month to <5 years.

**Outcome measures:**

Primary outcome—proportion of parents who reported use of the allocated study emollient every day for the duration of follow-up (12 weeks). Other feasibility outcomes—participant recruitment and retention, data collection and completeness and blinding of observers to allocation.

**Interventions:**

Aveeno lotion, Diprobase cream, Doublebase gel, Hydromol ointment.

**Results:**

197 children were recruited—107 by self-referral (mainly via practice mail-outs) and 90 by inconsultation (clinician consenting and randomising) pathways. Participants recruited inconsultation were younger, had more severe Patient-Oriented Eczema Measure scores and were more likely to withdraw than self-referrals. Parents of 20 (10%) of all the randomised participants reported using the allocated emollient daily for 84 days. The use of other non-study emollients was common. Completeness of data collected by parent-held daily diaries and at monthly study visits was good. Daily diaries were liked (81%) but mainly completed on paper rather than via electronic (‘app’) form. Major costs drivers were general practitioner consultations and eczema-related prescriptions. Observer unblinding was infrequent, and occurred at the baseline or first follow-up visit through accidental disclosure.

**Conclusions:**

It is feasible in a primary care setting to recruit and randomise young children with eczema to emollients, follow them up and collect relevant trial data, while keeping observers blinded to their allocation. However, reported use of emollients (study and others) has design implications for future trials.

**Trial registration number:**

ISRCTN21828118/EudraCT2013-003001-26.

**Strengths and limitations of this study**

- This pragmatic study demonstrates that it is feasible to recruit children with eczema from primary care, randomise them to a ‘leave on’ emollient and follow them up for 12 weeks with good observer blinding.
- Participant retention was better in participants who referred themselves into the study compared with those who were recruited during consultations with their general practitioner or practice nurse, although they also differed in respect of their age and parent-reported eczema severity.
- While it was possible to collect daily, weekly and monthly outcome data, missing data in parent-completed diaries have made interpretation of adherence to allocation challenging.
- There were practical and technical limitations with the ‘app’ version of the parent-completed diary.

## Introduction

Eczema (also referred to as atopic dermatitis or atopic eczema) affects around 20% of children in the UK. Incidence peaks in the first 2 years of life and decreases thereafter.^1^ It is characterised by dry and itchy skin, and it can have a significant impact on the quality of a child's life and their family.^2^

In countries with strong systems of primary care, such as the UK, the majority of children with eczema are diagnosed and managed by their family physician or general practitioner (GP) with emollients and topical corticosteroids. Emollients are recommended for the majority of patients and they are primarily used as a ‘leave on’ treatment to reduce eczema symptoms. Applied directly to the skin, emollients reduce water loss by occlusion and/or directly adding water to the dry outer layers of the skin. However, there are many products and formulations available (lotions, creams, gels and ointments) that vary in their consistency from ‘light’ to ‘heavy’. Despite claims from the manufacturers, evidence that any one is better than another is weak.

Two recent systematic reviews have highlighted a paucity of research to help guide clinicians and patients in their choice.^3^ ^4^ In summary, the field is characterised by a lack of good quality, randomised controlled trials (RCTs) directly comparing commonly used emollients, with medium-term to long-term data on clinically relevant outcomes. While undertaking this research represents unique challenges, such as the range of possible emollients to compare and the inability to mask users to emollients of very different consistencies (eg, lotion vs ointment), patients and clinicians have highlighted it as an important issue. In the recent James Lind Alliance eczema treatment research priority setting partnership, ‘Which emollients are the most effective and safe in treating eczema?’ emerged as one of the highest ranked uncertainties for further research.^5^

In order to address this uncertainty, we wanted to undertake an RCT of commonly prescribed emollients for the treatment of childhood eczema in primary care. However, the feasibility of being able to conduct such a trial was questionable, because of key issues such as whether parents/carers would be willing to be assigned and use a randomly allocated emollient for several months, and uncertainty about optimal methods of recruitment and data collection. Therefore, we conducted a trial to determine the feasibility of recruiting, retaining and collecting outcome data on young children with eczema in a primary care setting, to inform the design of a full trial.

## Methods

### Design, participants and interventions

Full details can be found in the protocol paper.^6^ In brief, COMET was a feasibility study of a pragmatic, observer-blinded, RCT to compare the clinical and cost-effectiveness of ‘leave on’ emollients in the treatment of children with eczema. Throughout this paper, we will use the term ‘parent’ to denote all carers/guardians with parental responsibility.

Between July 2014 and April 2015, participants were recruited in primary care (general practice) via two pathways: ‘self-referral’ (usually in response to a letter sent by their practice inviting them to take part) or ‘inconsultation’ (an approach during a surgery visit by GP/practice nurse (PN), who also received consent and undertook randomisation). GPs/PNs were asked to record all approaches to potentially eligible participants on a ‘recruitment log’. At the end of the study practices also undertook searches to identify the number of potentially eligible children who had at least one contact with the practice during the recruitment period.

To be eligible, children had to have eczema, be aged 1 month to under 5 years and not be known to be sensitive or allergic to any of the study emollients or their constituents. Participants were randomly allocated by a web-based system (1:1:1:1 ratio) to one of four emollients (Aveeno lotion 400 mL, Diprobase cream 500 g, Doublebase gel 500 g, Hydromol ointment 500 g) to use as their leave-on emollient with the directions to ‘Use twice daily and when required’. Study emollients were prescribed for the duration of the study by participants' GP surgeries and issued by pharmacies, as per usual care. The trial manager telephoned participants 1 week after randomisation to ensure that the allocated treatment had been received and started. All other care (appointments, prescriptions, referrals) was as per usual care. Research team members (‘observers’) undertaking the baseline and follow-up visits (but not clinicians, parents or participants) were blinded to emollient allocation.

Three key changes to the original protocol were implemented in the final 4 months of recruitment. First, the diagnostic criterion was relaxed from ‘doctor diagnosis of eczema’ to ‘diagnosed by a doctor or an appropriately qualified health care professional with oversight from a medically qualified doctor’. Second, the upper age limit was raised from under 3 years to under 5 years of age. Third, the number of practices was increased from 16 to 22. These additional practices were only asked to do the mail-out, not use the inconsultation recruitment pathway as well.

### Outcomes

Participants were followed-up for 12 weeks (84 days). During this time, study visits were scheduled to take place 28, 56 and 84 days after baseline and parents were asked to complete a daily diary (paper and electronic ‘app’ versions were offered). In addition, the primary care electronic medical records (EMR) were reviewed for the 3 months participants were in the study.

Data were collected on:

- use of study emollient and other eczema treatments (daily parent reported);
- eczema severity: weekly parent reported (Patient-Oriented Eczema Measure, POEM;^7^ parent global assessment) and monthly observer completed (Eczema Area Severity Index, EASI;^8^ Six Area, Six Sign Atopic Dermatitis, SASSAD;^9^ Three-Item Severity, TIS^10^) assessments;
- quality of life: Atopic Dermatitis Quality of Life (ADQoL)^11^ and Dermatitis Family Impact (DFI)^12^ (both monthly parent reported);
- skin hydration using a corneometer (see below) (monthly observer collected);
- eczema-related prescriptions and healthcare resource use (weekly parent reported and EMR review);
- eczema-related personal costs, parent time off work and child time away from school/day care (weekly parent reported).

Parents who withdrew from the study at any point were asked to complete a withdrawal questionnaire. At the end of the study, parents were asked to complete an exit questionnaire which included questions about their experience of taking part in the study.

The primary outcome of this feasibility study was the proportion of parents who reported use of the allocated study emollient every day for the duration of follow-up (12 weeks). Secondary outcomes were participant recruitment and retention, data collection and completeness (including health economic), and the extent to which the observers were kept blinded to the intervention. Outcome data itself and other feedback will be presented elsewhere.

### Corneometry

Skin hydration was measured at two sites on the body (antecubital fossa and forearm) using a corneometer (Corneometer CM825, Courage & Khazaka electronic GmbH, Cologne, Germany), in arbitrary units of 0–100, with a higher measurement representing greater hydration. Presented measurements were adjusted for ambient temperature and humidity, to give the prediction of what each measurement would have been had it been taken in the average conditions seen in the study; 22°C and 48.6% relative humidity. This adjustment was based on an equation estimated by regressing the corneometry measurements taken in the study on the corresponding temperature and humidity readings (see online supplementary appendix).

10.1136/bmjopen-2016-012021.supp1 supplementary appendix

### Sample size

Since this was a feasibility study, a formal sample size calculation was not required. We aimed for a target sample size of 160 participants. With this number, a true consent rate of 50% (160 children participating having invited 320 potentially eligible children) would be estimated with 95% CI of the order 44–56%.

### Analysis

We conducted linear or logistic regression (as appropriate) to compare the characteristics of participants recruited via the two recruitment pathways and those who withdrew/stayed in the study. Observer blinding was assessed using the Bang blinding index,^13^ which takes a value between −1 and +1: +1 indicates complete lack of blinding and 0 is consistent with perfect blinding. Negative values indicate the respondent is wrong more often than would be expected by chance, which can arise, for example, if all participants are said to be on one particular treatment irrespective of what they receive.

Healthcare resource use and prescribed medications were costed using relevant unit costs^14–17^ valued in pound sterling and at 2014 prices. The cost of the intervention emollients was estimated using three alternative methods; first, via the prescription cost analysis (PCA),^15^ second using the British National Formulary (BNF)^18^ and third using the Drug Tariff (DT)^19^ and Dictionary of Medicines and Devices (DMD).^20^ The final method aimed to estimate the true cost to the NHS of prescribed medications by estimating the amount community pharmacists are reimbursed for dispensing prescriptions. This method incorporates a deduction for any discount the pharmacy may have received, dispensing fees and payments for containers, consumables or other associated costs.

Health state utility values were estimated at each time point using scores from the ADQoL.^11^ QALYs were derived using the area under the curve approach^21^ and by multiplying to an annual equivalent.

### Ethics

The study was approved by Central Bristol Research Ethics Committee (REC reference: 13/SW/0297), Clinical Trial Authorisation given by the Medicines and Healthcare products Regulatory Agency (MHRA reference: 03299/0017/001-003) and research governance approvals obtained across all areas prior to the start of recruitment. Written informed consent was received from all participants.

## Results

### Recruitment of participants

**Figure 1:**
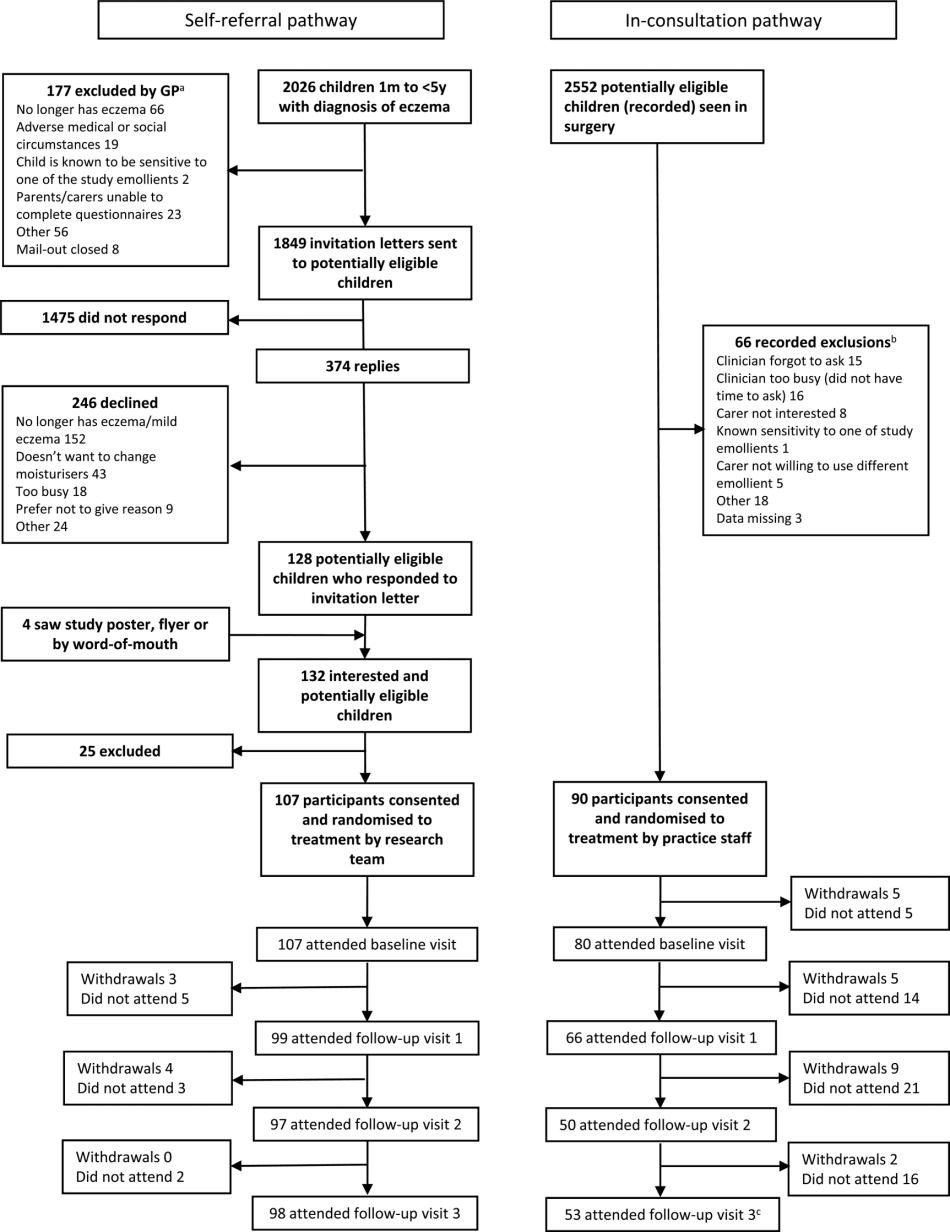
CONSORT diagram—recruitment by referral pathway’ with accompanying text: ^a^Data from 13 general practitioner (GP) practices (8 practices had no exclusions, 1 practice had 3 exclusions with no reasons given); ^b^data from 6 GP practices whose GPs returned recruitment logs; ^c^one participant withdrew after visit 3 from the inconsultation pathway.

Between July 2014 and May 2015, 197 children were recruited via 22 practices. The flow of the participants through the trial is shown in the CONSORT diagram (figure 1), which distinguishes between the two recruitment pathways.

Recruitment by self-referral pathway: 2026 potentially eligible children were screened and GPs excluded 9% (177/2026), the most common reason being ‘no longer has eczema’ (37%, 66/177). Of the 1849 invitation letters sent, responses were received from 20% (374/1849) with 66% (246/374) declining to take part. Again, the most common reason for not taking part was the child having either no or only mild eczema (62%, 152/246). A further 4 children were recruited by word-of-mouth or after seeing a study poster/being given a study flyer, giving a total of 132 potentially eligible participants who were screened by the research team. Of these, 19% (25/132) were not recruited, mainly because the carer could not be contacted (12/25, 48%).

Recruitment by inconsultation pathway: Retrospective searches identified 2552 potentially eligible children who had at least 1 contact with their practice, and therefore could have been approached via this pathway. Recording of these contacts by clinicians was poor, with only 6 practices returning ‘recruitment logs’, which detailed 66 encounters. Of these, the most common reasons given for not recruiting were that clinicians either forgot to ask or were too busy (47%, 31/66).

The majority of participants (62%, 54/87) recruited during the first 6 months came via the self-referral pathway (see online supplementary figure S1). However, during the final 4 months of recruitment, the number of inconsultation referrals increased so that by the end of the study, 90 (46%) of the total 197 participants came via this route. Practices 1–16 sent reminder letters to families who did not respond to the initial invitation and still met the eligibility criteria, which resulted in the recruitment of four additional participants.

10.1136/bmjopen-2016-012021.supp2 supplementary data

### Characteristics of participants

The mean age (SD) of participants at baseline was 21.7 months (12.8), with 85 (43%) female, 155 (85%) white and a mean Index of Multiple Deprivation (IMD) score (SD) of 21.8 (14.2) (generated from participant postcode). The mean (SD) eczema severity scores were as follows: POEM 8.8 (5.9), EASI 2.9 (3.8), SASSAD 8.8 (8.4) and TIS 2.0 (1.7). The mean (SD) DFI and ADQoL were 3.6 (4.8) and 0.787 (0.084), respectively.

However, as can be seen from table 1, participants recruited inconsultation were younger (mean age in months 17.0 vs 25.7, p<0.031), and had higher mean POEM (10.3 vs 7.6, p=0.012) scores than those who were recruited via self-referral.

**Table 1:** Characteristics of participants at baseline by referral pathway

	Self-referral (N=107)		Inconsultation (N=90)		p Value
		n		n	
Mean age in months (SD)	25.7 (11.6)	107	17.0 (12.6)	90	0.031*
Number female (%)	46 (43%)	107	39 (43%)	90	0.868†
Number white (%)	98 (93%)	108	57 (74%)	77	0.088†
Mean IMD score (SD)	15.7 (10.5)	104	25.4 (13.8)	88	0.201*
Mean eczema severity scores (SD)					
POEM (min 0, max 28, high=worse)	7.6 (5.7)	107	10.3 (5.8)	89	0.012*
EASI (min 0, max 72, high=worse)	2.8 (4.1)	105	3.1 (3.4)	79	0.841*
SASSAD (min 0, max 108, high=worse)	9.0 (8.7)	107	8.5 (7.9)	79	0.918*
TIS (min 0, max 9, high=worse)	2.1 (1.9)	107	2.0 (1.5)	79	0.571*
Skin hydration‡ (high=better)					
Forearm	31.3 (11.8)	98	32.9 (10.1)	70	0.719*
Antecubital fossa	36.5 (14.8)	98	39.5 (12.6)	71	0.325*
Mean DFI score (SD) (min 0, max 30, high=worse)	2.9 (4.0)	107	4.6 (5.6)	79	0.224*
Mean ADQoL (SD) (min 0.356, max 0.841, high=better)	0.799 (0.065)	105	0.770 (0.103)	75	0.239*

*Linear regression model adjusting for GP practice.

†Logistic regression model adjusting for GP practice.

‡Measurements adjusted to average study conditions of temperature (22°c) and humidity (48.6%) (model described in the Methods section/online supplementary appendix). Data presented in arbitrary units ((min 0, max 100, high=more hydrated).

### Participant retention

Twenty-eight (14%) participants withdrew from the study and 151 (77%) attended the final follow-up visit. Most participants who withdrew were recruited inconsultation (21/90, 23%, compared with 7/107, 7%, of self-referrals), including five children who did not attend their baseline visit. All bar one participant (who was recruited inconsultation) returned a withdrawal questionnaire, and the most commonly cited reason for withdrawing was lack of time (table 2).

**Table 2:** Reasons for participant withdrawal by recruitment pathway

Reasons for withdrawal*	Recruitment pathway	
	Self-referral (n=7)	Inconsultation (n=21)
Study emollient not working/effective	0	2
Adverse reaction to study emollient	2	0
Disliked emollient given	0	2
Just simply changed my mind	0	2
Do not have enough time	4	10
My child's skin has improved—no longer need emollient	0	4
Other	2	7

*More than one reason could be cited.

### Collection and completeness of outcome data

Twenty-two of 185 (12%) parents started using the app version of the daily diary, but only 11 people used it for the duration of the study. Technical problems meant that it was not promoted after the first 3 months of recruitment. Of 150 parents completing an exit questionnaire, 121 (81%) said they liked the daily diary, 22 (15%) said they were not sure and 7 (5%) disliked it.

Table 3 shows that completeness of daily, weekly and monthly data collected via the parent diary was generally good. However, completion rates for individual sections varied from 70% to 95% among those who returned the diaries and from 57% to 78% of all participants. The most poorly completed sections were daily record of eczema treatment use and weekly time off school and work. Owing to the cumulative nature of healthcare costs, complete costing of healthcare resources was possible for only 62% (122/197) of participants, despite the relevant section of each diary having been completed by at least 70% (138/197).

**Table 3:** Completeness of data collected by parent-completed daily diary

Frequency of question item completion	Question items	Diary 1 (days 1–28)			Diary 2 (days 29–56)			Diary 3 (days 57–84)		
		n	% of returners (n=162)	% of participants (n=197)	n	% of returners (n=151)	% of participants (n=197)	n	% of returners (n=150)	% of participants (n=197)
Daily	Eczema treatments	113	70	57	129	85	65	128	85	65
Weekly	POEM	145	90	74	139	92	71	139	93	71
	HCP contacts	141	87	72	138	91	70	138	92	70
	Time off school and work	130	80	66	114	75	58	124	83	63
Monthly	ADQoL	150	93	76	135	89	69	140	93	71
	DFI	153	94	78	141	93	72	143	95	73

ADQoL, Atopic Dermatitis Quality of Life; DFI, Dermatitis Family Impact; HCP, Healthcare Professional; POEM, Patient-Oriented Eczema Measure.

Completeness of data collected by the observers was also good, with the median number (IQR) of visits with complete data (maximum 4) for EASI, TIS and SASSSAD all being 4.0 (3.0 to 4.0). The completeness for corneometry was lower and differed by site (forearm 3.0 (2.0–4.0), antecubital fossa 4.0 (2.0–4.0)), as it was not possible to use the one available corneometer at concurrent follow-up visits. A greater proportion of observer visits occurred ±10 days than ±5 days of the scheduled date, and baseline visits were more likely to be timely for participants who self-referred (see online supplementary table S1).

The Bang blinding indices for observer unblinding to the different emollients are shown in table 4 (observer guess and treatment assignment for each assessment, by which this index was calculated, are shown in online supplementary tables S2–S5). Observers reported not knowing which study emollient the participants were using at most visits. They correctly identified the study emollient in eight participants at the baseline and first follow-up visits. The most common reasons given for unblinding were the parent telling them (6/8 baseline and 6/8 visit 1) or the observer seeing the study emollient during the visit (2/8 baseline and 1/8 visit 1).

**Table 4:** Bang blinding index with 95% two-sided CI (comparing correct treatment response with incorrect treatment or do not know response)

Study emollient	Visit			
	Baseline	1	2	3
Aveeno lotion	0.2 (0.0, 0.4)	0.0 (0.0, 0.1)	CE	CE
Hydromol ointment	0.1 (−0.1, 0.2)	0.0 (−0.1, 0.1)	CE	CE
Diprobase cream	0.1 (0.0, 0.2)	0.1 (0.0, 0.2)	CE	CE
Doublebase gel	0.1 (0.0, 0.2)	0.0 (−0.1, 0.0)	CE	CE

CE, cannot be estimated due to lack of data (see online supplementary tables S2–S5).

### Adherence to intervention

Of the 197 participants, parents of 20 (10%) reported using their allocated emollient daily for 84 days (primary outcome). However, 38 (19%) did not give any data on study emollient use. Therefore, of the 159 parents of participants who completed this question at least once, 49 (31%) said they used the study emollient on each occasion that they completed the question.

The majority (156/162, 96%) of parents reported some use of a non-study emollient: 25% (41/162) reported use every day (that data were provided) and 51% (82/162) reported using an ‘other’ emollient on up to 50% of days. Fifty-three per cent (85/159) of parents reported using an ‘other’ emollient *instead* of the study emollient at least once. Of these 85 parents, 86% (73/85) used an ‘other’ emollient rather than the study emollient half of the time.

Analysis of the EMRs revealed that while the mean (SD) number of intervention emollient prescriptions was 1.44 (0.78), the number of eczema-related prescriptions for non-intervention emollients was 1.28 (2.13). This included a mean (SD) number of 0.48 (0.99) non-study emollients, 0.43 (0.93) topical corticosteroid and 0.31 (0.69) bath/shower product prescriptions.

### Economic evaluation

The main cost driver as recorded in the EMRs was GP appointments with a mean cost of £11.77 over the follow-up period (see online supplementary table S6). On average, the cost of eczema-related prescribed medications excluding trial emollients equated to £6.97. Healthcare and intervention emollient costs did not appear to differ considerably between treatment arms (table 5). The mean (SD) cost to the NHS of each of the trial emollients using the DT and DMD was £11 (£5), £9 (£5), £9 (£5) and £8 (£5), for Aveeno lotion, Diprobase cream, Doublebase gel and Hydromol ointment, respectively. Emollient costs were slightly higher using this method in comparison to the PCA or BNF (table 5). Complete ADQoL data for 119 participants allowed estimation of mean (SD) annual QALYs of 0.799 (0.061).

**Table 5:** Total healthcare cost (£) and QALYs, by treatment allocation

	Aveeno lotion (N=51)			Diprobase cream (N=53)			Doublebase gel (N=46)			Hydromol ointment (N=47)		
	n	Mean cost	SD	n	Mean cost	SD	n	Mean cost	SD	n	Mean cost	SD
Total healthcare cost—EMR only	51	23	(50)	53	28	(50)	46	32	(85)	47	62	(258)
Total healthcare cost—EMR plus diary	30	25	(97)	32	32	(54)	33	35	(93)	27	16	(21)
Intervention emollient cost (PCA)	51	8	(4)	53	9	(5)	46	9	(5)	47	7	(4)
Intervention emollient cost (BNF)	51	8	(4)	53	9	(5)	46	8	(4)	47	7	(4)
Intervention emollient cost (DT and DMD)	51	11	(5)	53	9	(5)	46	9	(5)	47	8	(5)
Total cost (EMR, diary and DT and DMD emollient)	30	38	(98)	32	42	(57)	33	43	(94)	27	24	(23)
Annual QALYS	32	0.798	(0.061)	29	0.812	(0.055)	33	0.790	(0.061)	25	0.800	(0.070)

Parents reported additional expenditure due to their child's eczema on items including: eczema treatments (n=25), clothes (n=14), household items (n=12), toiletries (n=11) and food and drink (n=9). Time off paid employment or school/day care was reported infrequently: over the entire follow-up period, only 3 days off paid employment and 2 days off school/day care were reported across all participants.

## Discussion

This is the first study to show that it is feasible in a primary care setting to recruit and randomise young children with eczema to ‘leave-on’ emollients and follow them up, keeping observers blinded to their allocation. We exceeded our recruitment target, although in the final 4 months, we enlisted the help of six more practices than originally planned and relaxed the age and diagnostic eligibility criteria. Reported daily use of the study emollients was low, however, and the use of other emollients was common.

We conducted a well-executed, pragmatic trial overcoming many practical and logistical challenges, meeting regulatory requirements of a Controlled Trial of an Investigational Medicinal Product (CTIMP). More detail on trial conduct can be found in the published trial protocol^6^ and we report the findings in accordance with the CONSORT guidelines.^22^ These findings have implications for future trials of emollients and other treatments for children with eczema, but also for trials of treatments of other long-term conditions in primary care with medium-term follow-up.

A strength of this study is its exploration of the two possible recruitment pathways and their feasibility in a main trial. By asking most practices to try and recruit via the two routes, we now have a strong understanding of the number (and characteristics) of children likely to be recruited in a definitive trial, and the proportion likely to withdraw (and reasons why). While having the two pathways into the study helped the trial meet its recruitment target, we are mindful that the characteristics of participants, and their commitment to staying in the trial differed for participants recruited via these two routes. Of 90 participants recruited via the inconsultation pathway, 21 (23%) withdrew and 53 (59%) attended their final appointment, compared with 7 (7%) and 98 (92%), respectively, for participants recruited via self-referral (most mail-out). With respect to the mail-out invitation, the high number of children identified with mild or no eczema reflects the fact that for many, their diagnosis will be historical and for others, erroneous. One way to improve this may be to limit invitations to children with a recent relevant prescription (suggesting ‘active’ disease), as per the BATHE study.^23^ The rise in the rate of children recruited inconsultation may reflect both the staggered nature in which the practices came into the study but also a learning and confidence effect among recruiting clinicians. Although all practices were members of the Clinical Research Network, they had variable levels of experience in recruiting to studies of this type and each study has its unique processes that have to be followed.

In addition to investigating recruitment and retention, we have also collected important adherence, outcome (including corneometry) and health economic data. We found that it is feasible to collect and cost the data required to perform an economic evaluation in this setting. EMR records provided a rich source of complete healthcare resource use data, indicating that in further studies, healthcare resource use collected from diaries could be reduced. Given that time away from paid employment and school were very rarely reported, capturing these data in a future trial would likely be less important. Our assessment of additional items bought due to eczema has highlighted a list of important categories to include in future studies. At inception, no generic measure of health-related quality of life that was psychometrically and conceptually robust enough for young children under the age of 3 was available. For this reason, we used the ADQoL, from which we were able to estimate QALYs. However, given that this is a condition-specific preference-based measure, the results may not be comparable across conditions.

Reported use of study emollients was low and use of other emollients either alongside or instead of the allocated treatment common, but our ability to interpret these findings is limited by missing data. While completion of the daily diary was generally good, questions on the use of eczema treatments (including study emollient) were the most poorly completed. We think this is because parents left an item blank when a treatment was not used, rather than recording ‘None’ or ‘0’, meaning it was classed as ‘missing’. For example, if missing data on ‘other’ emollient use are treated as ‘no use’ on those days for which complete data are available on the use of the study emollient, then only 6% (10/162) (compared with 25%) of parents of participants used a non-study emollient every day and 71% of parents of participants (115/162) (compared with 51%) reported using another emollient on up to 50% of days. Another limitation in the data on emollient use is that we are unable to distinguish between use as leave-on therapy (in the same way as the study emollients) and use as soap substitute. Missing data would have been less of a problem had the ‘app’ version of the diary worked better and been used by more parents—one of the attractions of collecting data from parents this way is the ability to automatically monitor data entry in real time and prompt parents to answer all the questions.

Although initial interest among parents in using our ‘app’ was high, its development and incorporation in this study was challenging because: (1) regulatory requirements for CTIMPs meant, additional and time-consuming testing to ensure that data were transmitted securely; and (2) the cost required to develop a fully functional app for the most common smart phone and tablet platforms (iOS and Android). Therefore, while we cannot conclude that studies similar to ours should not consider data collection via bespoke apps, we would certainly caution against underestimating the time, cost and technical implications of doing this. In future studies, automatic email prompts to parents to complete online questionnaires are probably a safer and more cost-effective way of maximising data collection. For parents who would still prefer paper questionnaires, clear instructions should be given about the importance of positively indicating ‘no treatment use’ (as opposed to leaving an answer blank). We also recommend that future studies involving young children should be realistic about parents' ability to adhere to strict follow-up schedules, with study protocols providing realistic ‘windows’ (eg, ±10 days if permissible) within which to expect data collection; and where it is desirable to keep the observer blinded, giving unambiguous instructions to parents to avoid accidental disclosure.

For future trials comparing emollients, researchers should be encouraged that participants who consent to taking part generally stay in the trial, although they may wish to recruit participants using just the self-referral pathway. Researchers also need to be aware that couse of emollients appears to be common, and the extent to which this matters will depend on where future studies sit on the efficacy–effectiveness spectrum. Whatever the design, this study provides the foundations for future definitive studies to answer the prioritised research question ‘Which emollient is the most effective and safe in treating eczema?’^5^
