# Supplementary material for: Choice of Moisturiser for Eczema Treatment (COMET): feasibility study of a randomised controlled parallel group trial in children recruited from primary care
Source: BMJ Open. 2016 Nov 16;6(11):e012021. doi: 10.1136/bmjopen-2016-012021 (PMC5129109; doi:10.1136/bmjopen-2016-012021)
Supplement: supplementary appendix [file bmjopen-2016-012021supp_appendix.pdf]

Choice of Moisturiser for Eczema Treatment (COMET): feasibility study of randomised controlled parallel group trial in children recruited from primary care

Appendix: Skin hydration multivariable model

Skin hydration is affected by room temperature and humidity and all measurements were undertaken in participant's homes, in non-standardised conditions. We did not identify any published guidance on an analysis method to account for these environmental influences on the readings, so we constructed a multivariable linear regression model to adjust for the variation in temperature and humidity. Coefficients for this model were estimated by regressing the mean of the three measurements at baseline in each of the two sites (antecubital fossa and forearm) as the outcome on the temperature and humidity of the room. This model (calculation A below) predicted an average skin hydration measurement of 34.875 for the study average conditions of temperature (22 degrees centigrade) and humidity (48.6 units). The difference between the observed level of hydration, and the average level of hydration predicted for the temperature and humidity in which measurements were taken (the residual, calculation B) was added to the constant of 34.875 (calculation C) giving the skin hydration measure adjusted to average conditions of temperature and humidity. These calculations were done for each visit and for each site (antecubital fossa and forearm):

A: Predicted outcome model: Predicted average skin hydration= 2.452 + 1.177* temperature + 0.135* humidity

B: Residual = Actual measurements taken-Predicted average skin hydration

C: Adjusted measure of skin hydration (corneomtry outcome) = 34.875 + Residual
