# Supplementary material for: Choice of Moisturiser for Eczema Treatment (COMET): feasibility study of a randomised controlled parallel group trial in children recruited from primary care
Source: BMJ Open. 2016 Nov 16;6(11):e012021. doi: 10.1136/bmjopen-2016-012021 (PMC5129109; doi:10.1136/bmjopen-2016-012021)
Supplement: supplementary data [file bmjopen-2016-012021supp.pdf]

Choice of Moisturiser for Eczema Treatment (COMET): feasibility study of randomised controlled parallel group trial in children recruited from primary care

Supplementary figure & tables

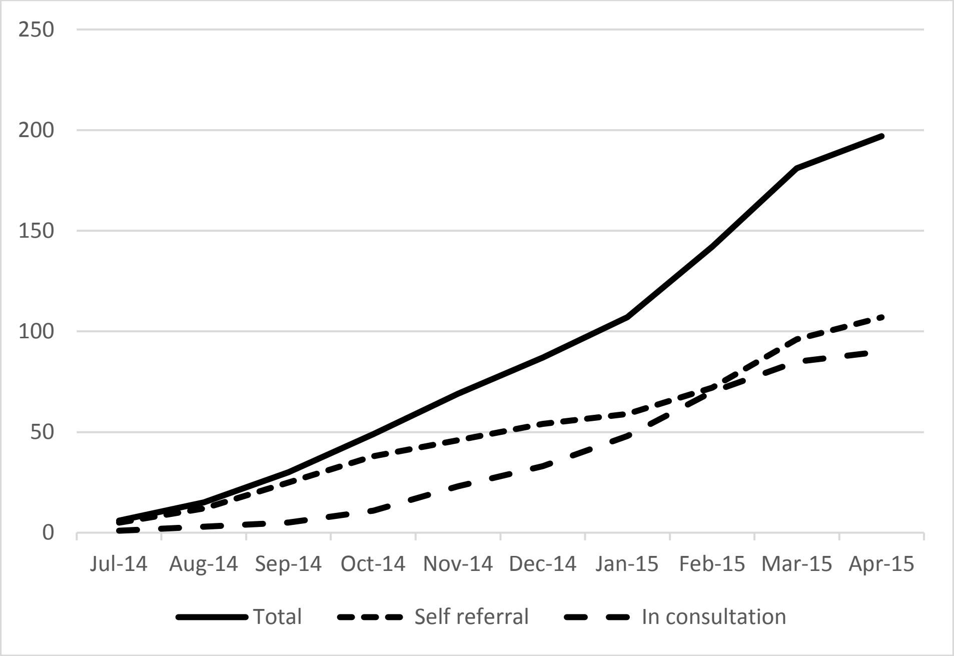

Figure S1: Cumulative participant recruitment: total and by referral pathway

	n/N (%)				
Appointment window	±5 days		±10 days		Number of assessments with complete date data‡
Recruitment pathway	Self-referral	In-consultation	Self-referral	In-consultation	
Baseline†	102/107 (95)	26/80 (33)	104/107 (97)	64/80 (80)	187
Visit 1	72/99 (73)	55/68 (81)	99/99 (100)	68/68 (100)	167
Visit 2	75/97 (77)	41/51 (80)	94/97 (97)	49/51 (96)	148
Visit 3	67/97 (69)	39/49 (80)	90/97 (93)	49/49 (100)	146

† Baseline visit fell within +/- 5/10 days of referral date

‡ Missing date of assessment or referral date possible

Table S1: Number (proportion) of researcher baseline and follow-up visits taking place within ±5 and ±10 days of planned scheduled dates by recruitment pathway

	Researcher guess					
Assignment	Aveeno® cream	Hydromol® ointment	Diprobase® cream	Doublebase® gel	Don't know	Total
Aveeno® cream	3	0	0	0	14	17
Diprobase® cream	0	0	2	0	20	22
Doublebase® gel	0	0	0	2	18	20
Hydromol® ointment	0	1	0	0	16	17
Total	3	1	2	2	68	76

The 109 participants who self-referred were prescribed their study emollient after the baseline visit; and 1 participant was recorded as an "other" response.

Table S2: Number of subjects by treatment assignment and guess at baseline visit

	Researcher guess					
Assignment	Aveeno® cream	Hydromol® ointment	Diprobase® cream	Doublebase® gel	Don't know	Total
Aveeno® cream	1	0	0	0	37	38
Diprobase® cream	0	0	3	1	39	43
Doublebase® gel	0	0	1	0	37	38
Hydromol® ointment	0	1	0	1	33	35
Total	1	1	4	2	146	154

CSOs recorded 5 participants as "other" response.

Table S3: Number of subjects by treatment assignment and guess at visit 1

	Researcher guess					
Assignment	Aveeno® cream	Hydromol® ointment	Diprobase® cream	Doublebase® gel	Don't know	Total
Aveeno® cream	1	0	0	0	36	37
Diprobase® cream	0	0	0	0	39	39
Doublebase® gel	0	0	0	0	36	36
Hydromol® ointment	0	1	0	0	30	31
Total	1	1	0	0	141	143

CSOs recorded 3 participants as other responses.

Table S4: Number of subjects by treatment assignment and guess at visit 2

	Researcher guess					
Assignment	Aveeno® cream	Hydromol® ointment	Diprobase® cream	Doublebase® gel	Don't know	Total
Aveeno® cream	0	0	0	0	39	39
Diprobase® cream	0	0	0	0	38	38
Doublebase® gel	0	0	0	0	38	38
Hydromol® ointment	0	0	0	0	32	32
Total	0	0	0	0	147	147

CSOs recorded 2 participants as other responses.

Table S5: Number of subjects by treatment assignment and guess at visit 2

EMR resource use for all participants (N=197)				
	Mean no contacts	(SD)	Mean cost	(SD)
GP face-to-face	0.31	(0.71)	11.77	(27.16)
GP telephone	0.10	(0.36)	2.22	(8.24)
GP out of hours	0.02	(0.12)	1.04	(8.39)
Nurse face-to-face	0.08	(0.27)	0.92	(3.11)
Nurse telephone	0.02	(0.17)	0.11	(0.91)
Other/unknown	0.04	(0.21)	10.63	(116.20)
Outpatient appointments	0.02	(0.12)	2.29	(20.06)
Prescribed medications	-		6.97	(12.05)

Table S6 Mean health care contacts and costs (£) from electronic medical records
